# Supplementary material for: MSF Enhances Human Antimicrobial Peptide β-Defensin (HBD2 and HBD3) Expression and Attenuates Inflammation via the NF-κB and p38 Signaling Pathways
Source: Molecules. 2023 Mar 18;28(6):2744. doi: 10.3390/molecules28062744 (PMC10058637; doi:10.3390/molecules28062744)
Supplement: Supplementary file 1 [file molecules-28-02744-s001.zip › molecules-2217482-supplementary.pdf]

Supplementary Materials

MSF Enhances Human Antimicrobial Peptide β-Defensin (HBD2 and HBD3) Expression and Attenuates Inflammation via the NF-κB and p38 Signaling Pathways

Anh-Thu Nguyen, Minho Kim, Ye-Eun Kim, Hangeun Kim, Sanghyun Lee, Yunji Lee and Ki-Young Kim

A

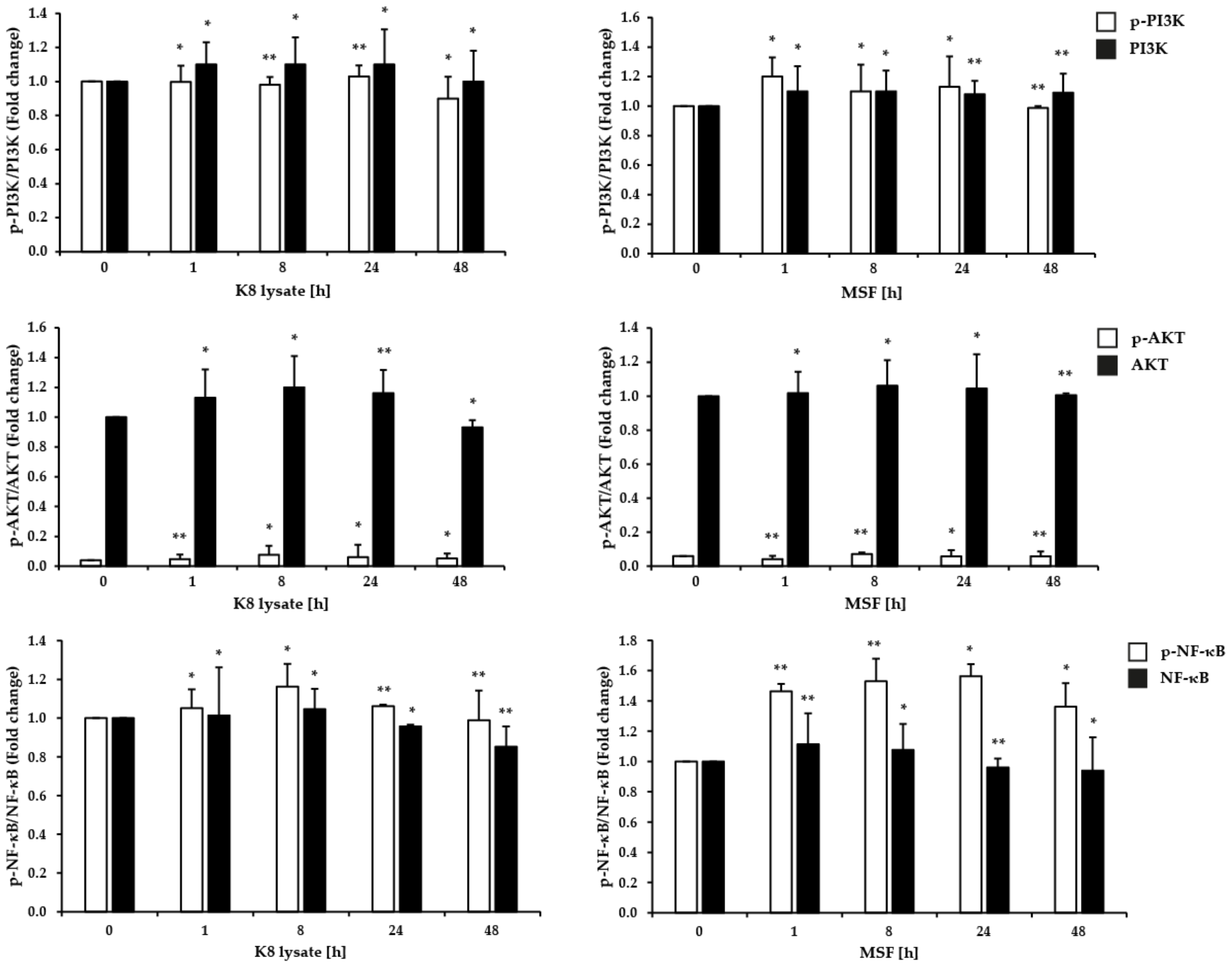

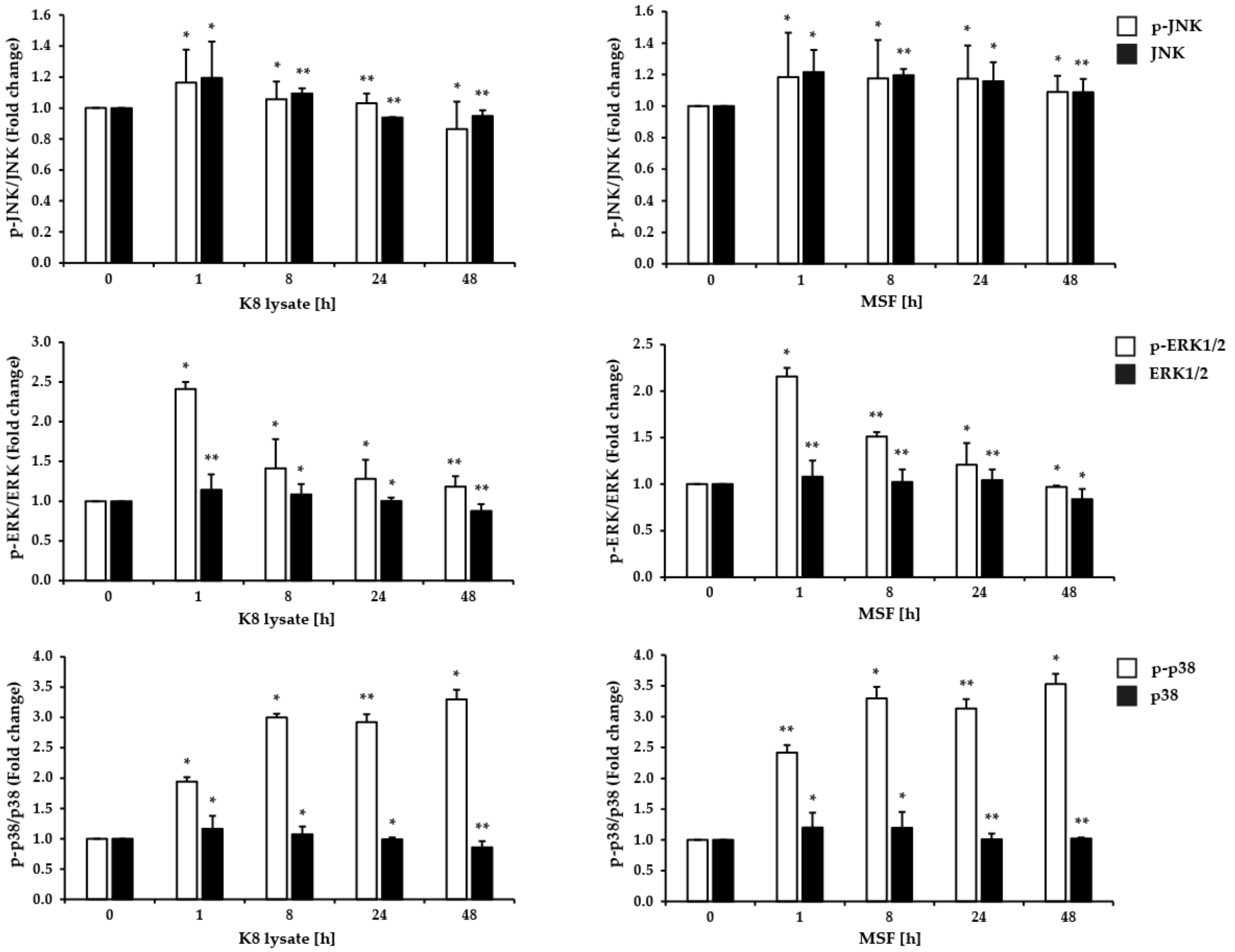
p-JNK/JNK (Fold change)
K8 lysate [h]
MSF [h]
p-JNK
JNK
p-ERK/ERK (Fold change)
p-ERK1/2
ERK1/2
p-p38/p38 (Fold change)
p-p38
p38

B

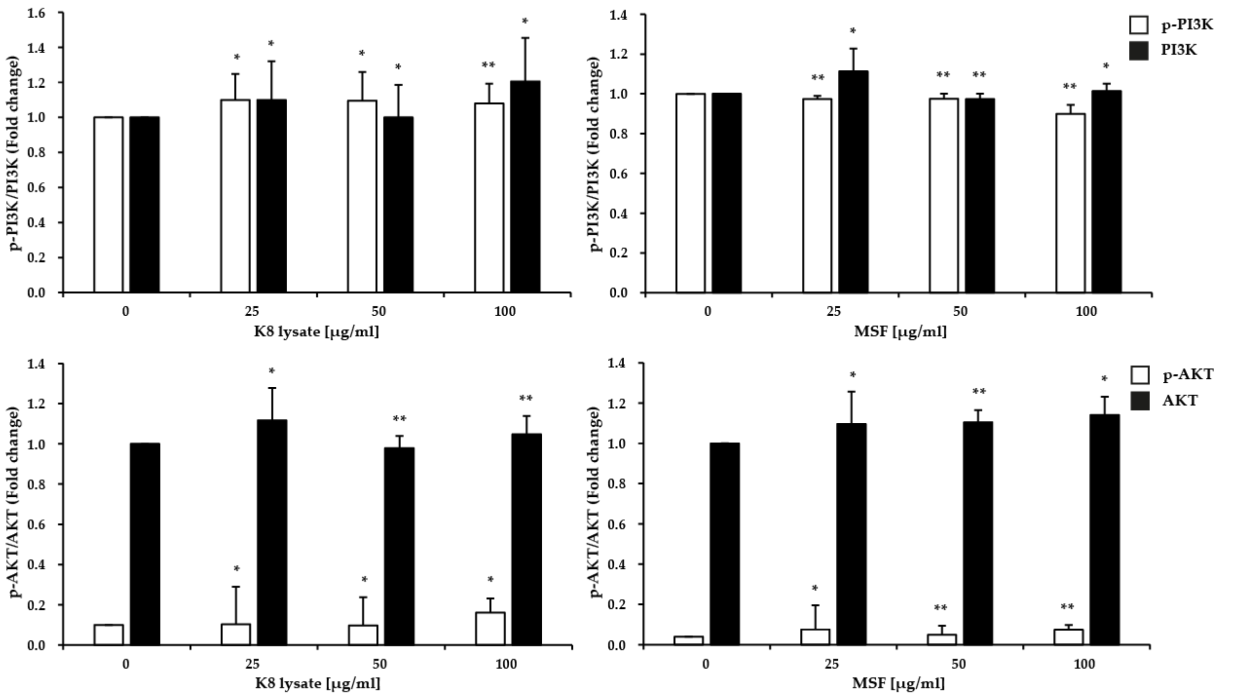
p-PI3K/PI3K (Fold change)
K8 lysate [µg/ml]
MSF [µg/ml]
p-PI3K
PI3K
p-AKT/AKT (Fold change)
p-AKT
AKT

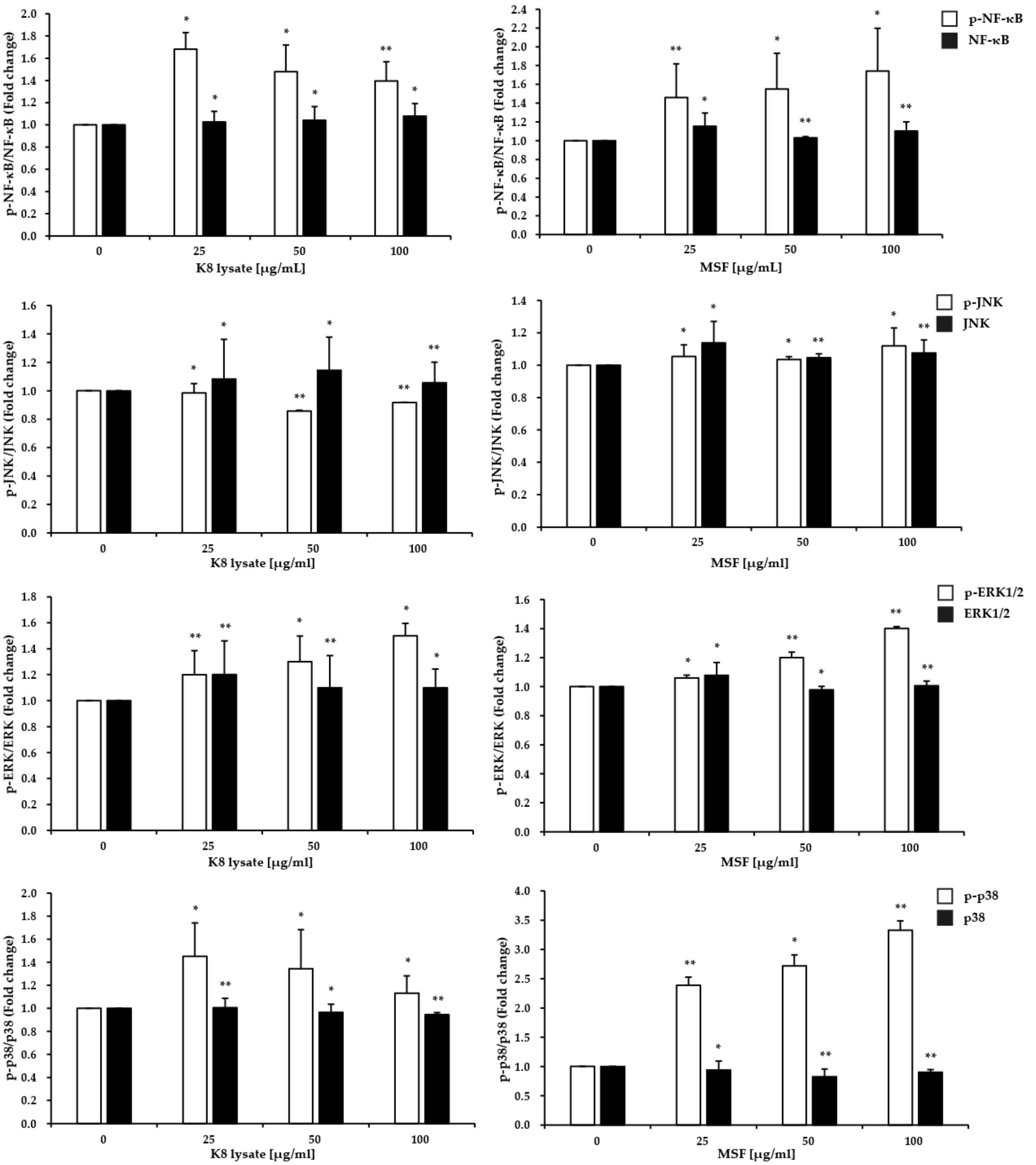

p-NF-κB/NF-κB (Fold change)
K8 lysate [μg/mL]
MSF [μg/mL]
p-NF-κB
NF-κB
p-JNK/JNK (Fold change)
K8 lysate [μg/ml]
MSF [μg/ml]
p-JNK
JNK
p-ERK/ERK (Fold change)
p-ERK1/2
ERK1/2
p-p38/p38 (Fold change)
p-p38
p38
0
25
50
100

C

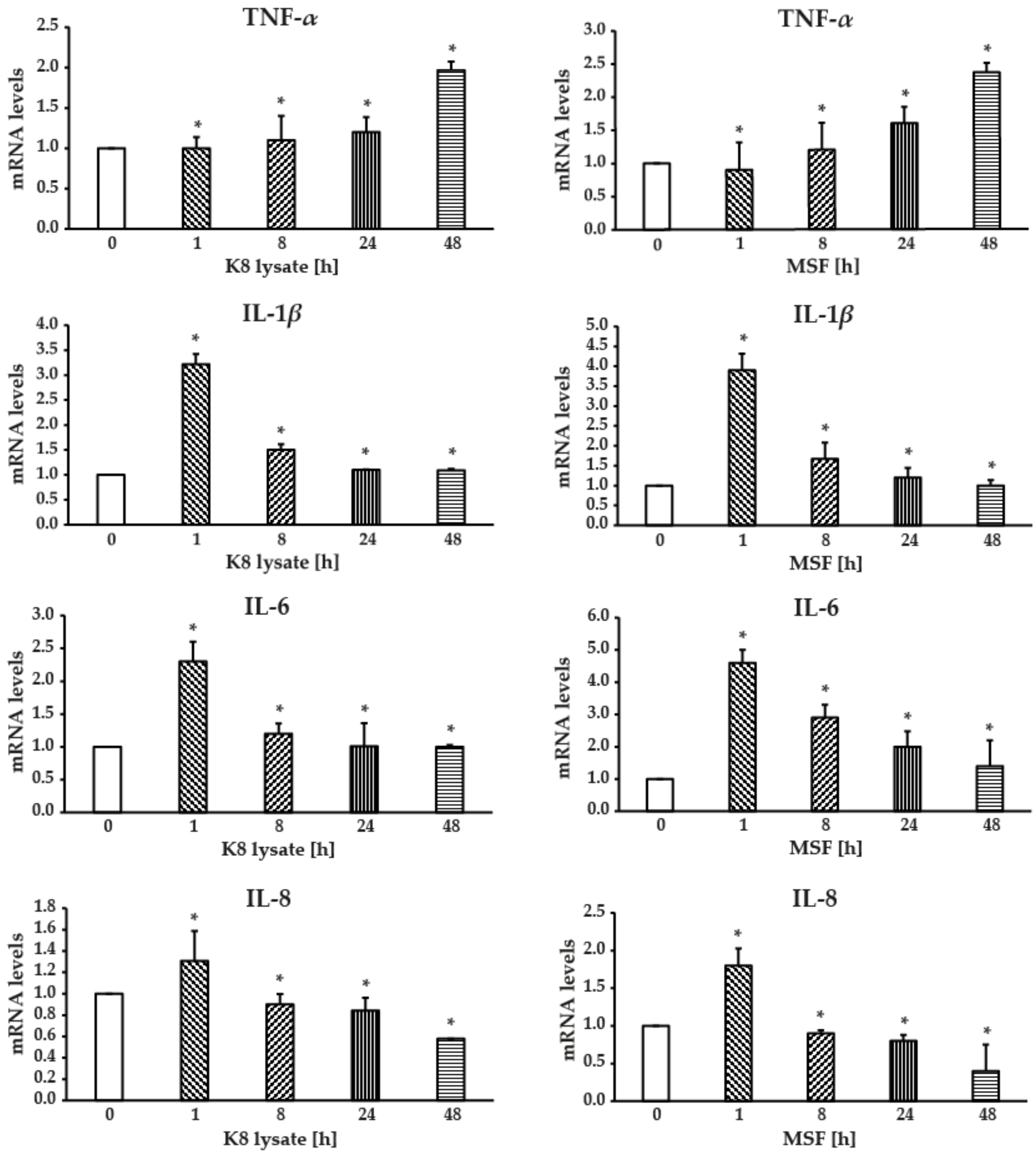
TNF-α
mRNA levels
2.5
2.0
1.5
1.0
0.5
0.0
0
1
8
24
48
K8 lysate [h]
TNF-α
3.0
2.5
2.0
1.5
1.0
0.5
0.0
MSF [h]
IL-1β
4.0
3.5
3.0
2.5
2.0
1.5
1.0
0.5
0.0
K8 lysate [h]
IL-1β
5.0
4.5
4.0
3.5
3.0
2.5
2.0
1.5
1.0
0.5
0.0
MSF [h]
IL-6
3.0
2.5
2.0
1.5
1.0
0.5
0.0
K8 lysate [h]
IL-6
6.0
5.0
4.0
3.0
2.0
1.0
0.0
MSF [h]
IL-8
1.8
1.6
1.4
1.2
1.0
0.8
0.6
0.4
0.2
0.0
K8 lysate [h]
IL-8
2.5
2.0
1.5
1.0
0.5
0.0
MSF [h]

D

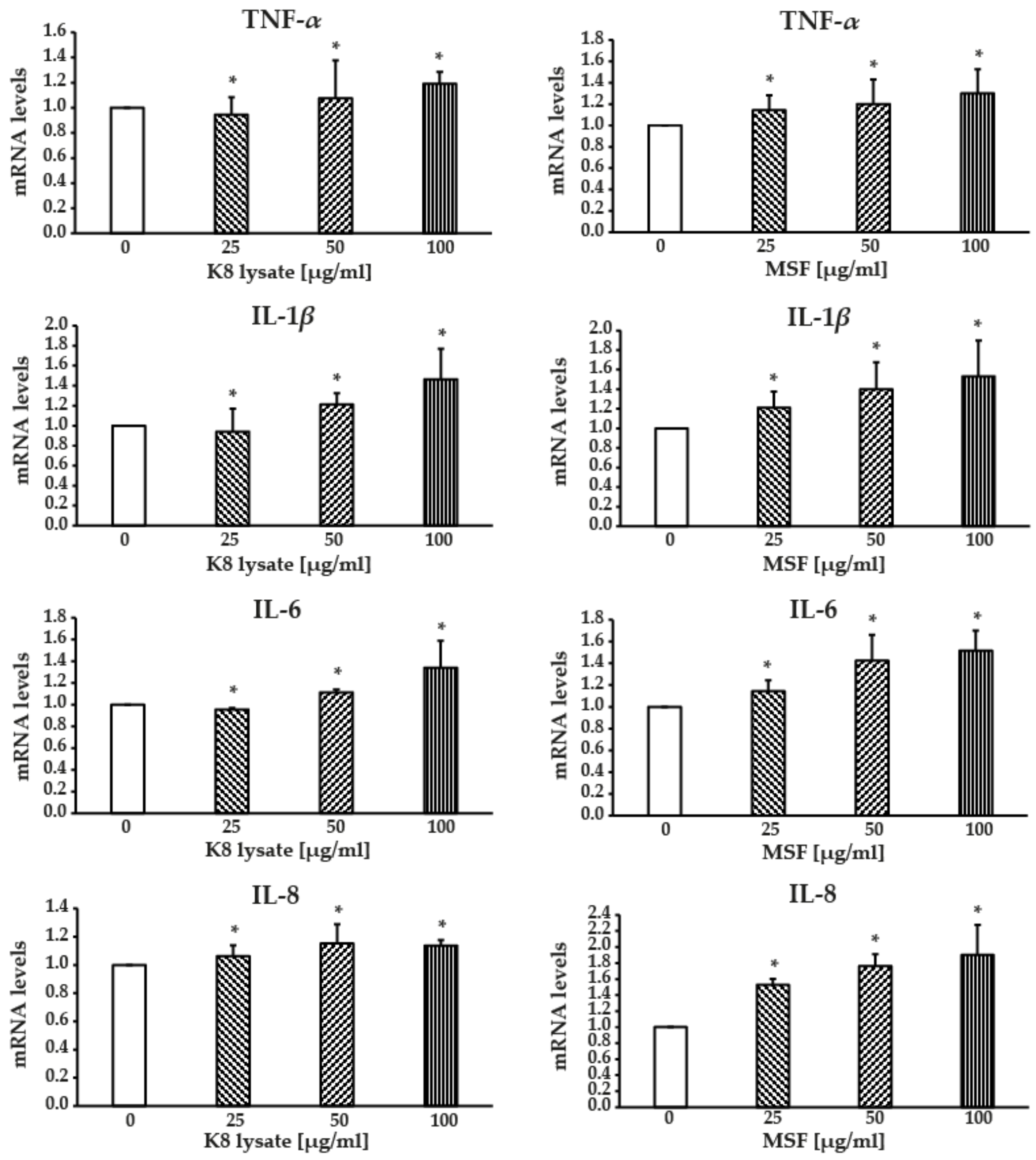

Figure S1. Effect of MSF treatment in PI3K, NF-κB, and MAPK signaling pathways. HaCaT cells were starved for 20 h before treatment of indicated concentration of MSF and K8 lysate for 1 h. (A and B) Western blot quantification of protein levels of PI3K, AKT, NF-κB, JNK, ERK1/2 and p38. (C and D) The mRNA expression of TNF-α, IL-1β, IL-6, and IL-8 by treatment of MSF and K8 lysate in a time- and dose- dependent manner. Values mean ± SD with three independent experiments. *$p < 0.05$, **$p < 0.01$.

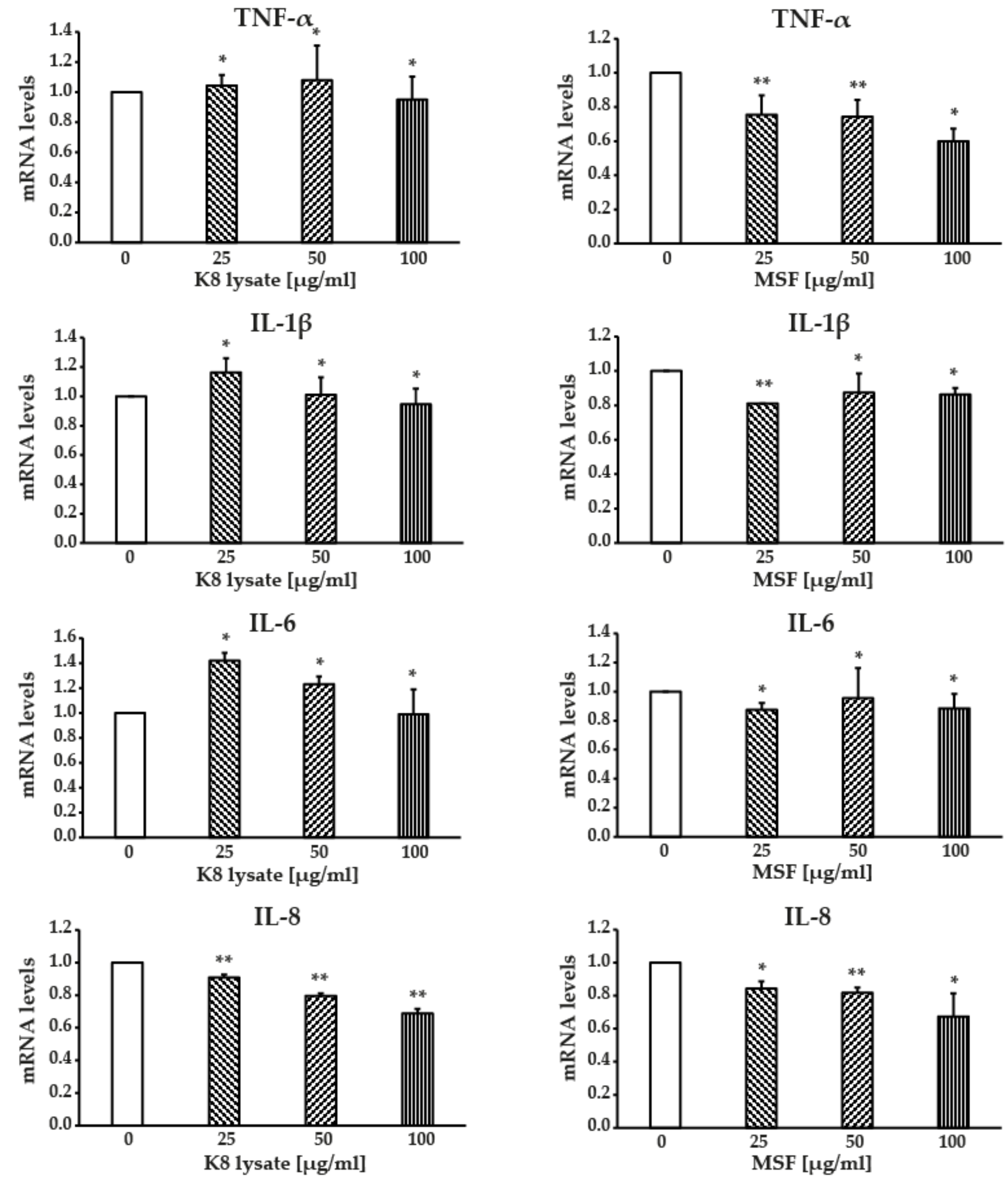

Figure S2. MSF treatment regulated the mRNA expression of pro-inflammatory cytokine genes via p38 signaling pathways. After 20 h starvation, cells were preincubated with SB203580, a specific inhibitor of p38, for 3 h and then treated indicated concentration of MSF for 1 h. The alteration of mRNA levels of TNF-α, IL-1β, IL-6 and IL-8 was analyzed using qRT-PCR. Values mean ± SD with three independent experiments. *$p < 0.05$, **$p < 0.01$.

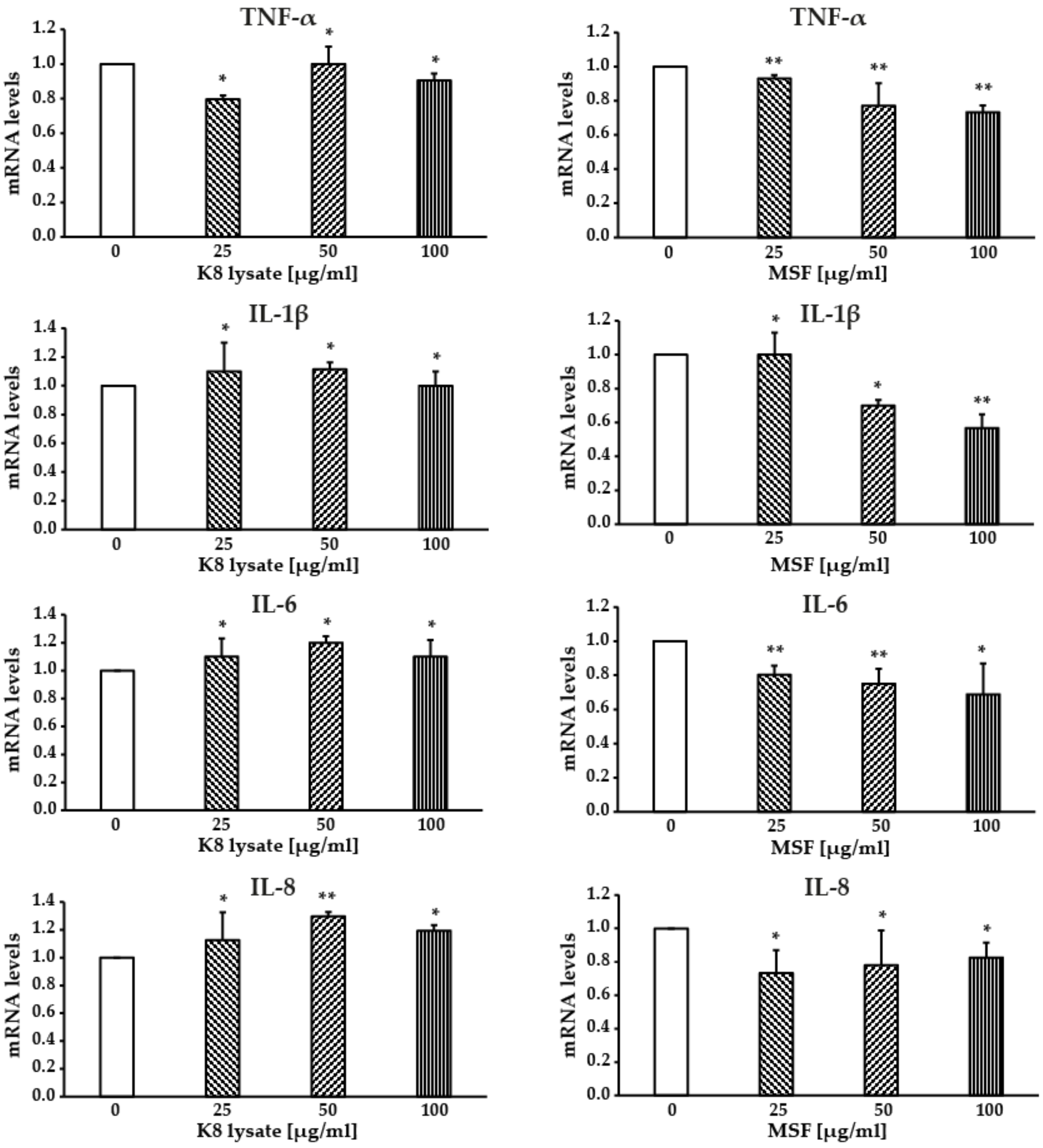

Figure S3. MSF regulated the expression of pro-inflammatory genes (TNF-α, IL-1β, IL-6 and IL-8) through NF-κB signaling pathways. HaCaT cells were starved for 20 h, and then were pre-treated with SP100030 (50 μM) for 3 h. The indicated concentration (25, 50, and 100 μg/ml) of MSF and K8 lysate were treated for 1 h. The expressions of TNF-α, IL-1β, IL-6 and IL-8 was checked by qRT-PCR. Values mean ± SD with three independent experiments. *$p < 0.05$, **$p < 0.01$.

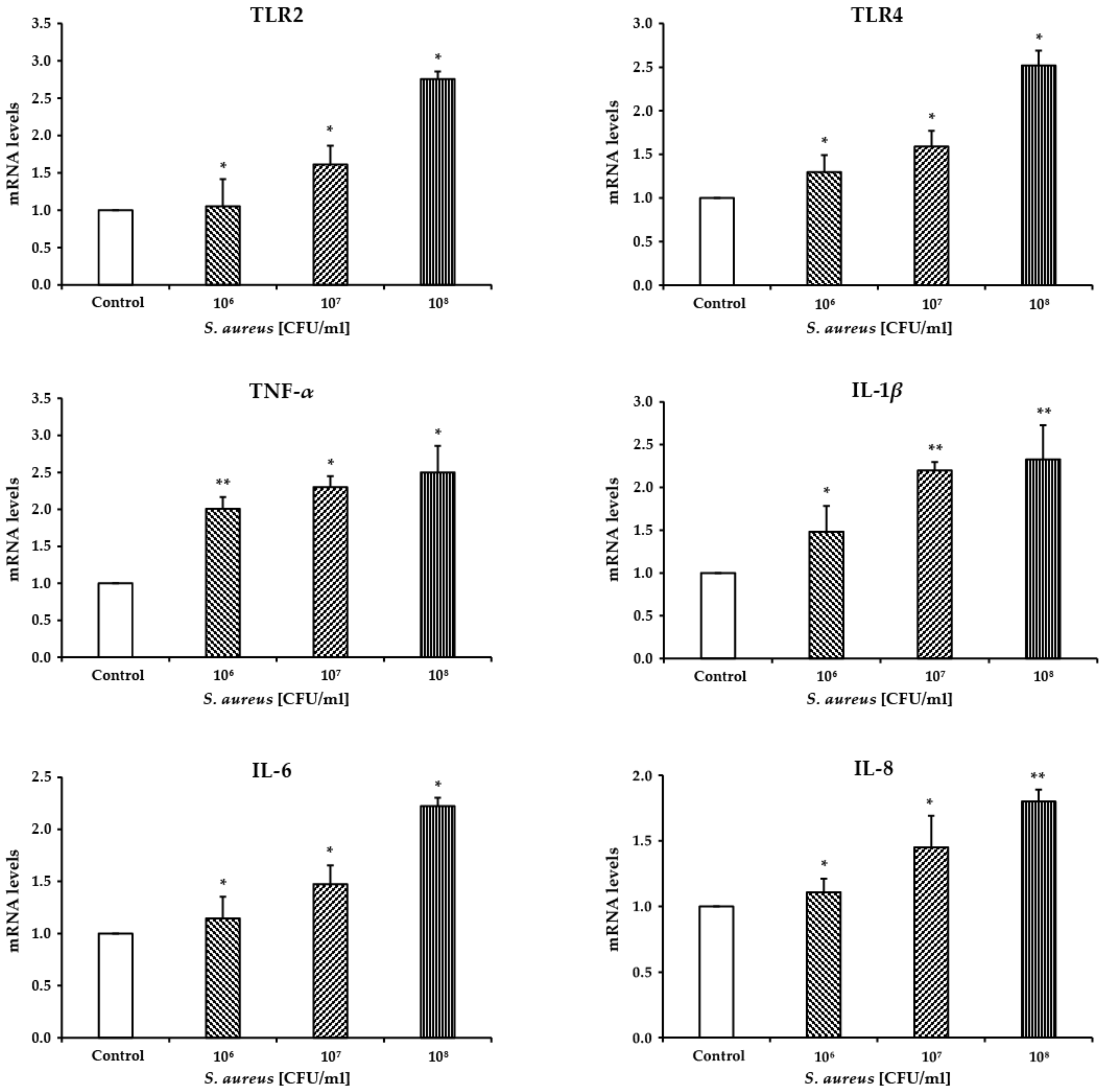

Figure S4. *S. aureus* increased the expression of TLR2, TLR4 and pro-inflammatory cytokines. Heat-killed *S. aureus* was treated to THP1 cells for 24 h. qRT-PCR was used to check the expression of TLR2, TLR4 and pro-inflammatory cytokines (TNF-α, IL-1β, IL-6 and IL-8). Values mean ± SD with three independent experiments. *$p < 0.05$, **$p < 0.01$.

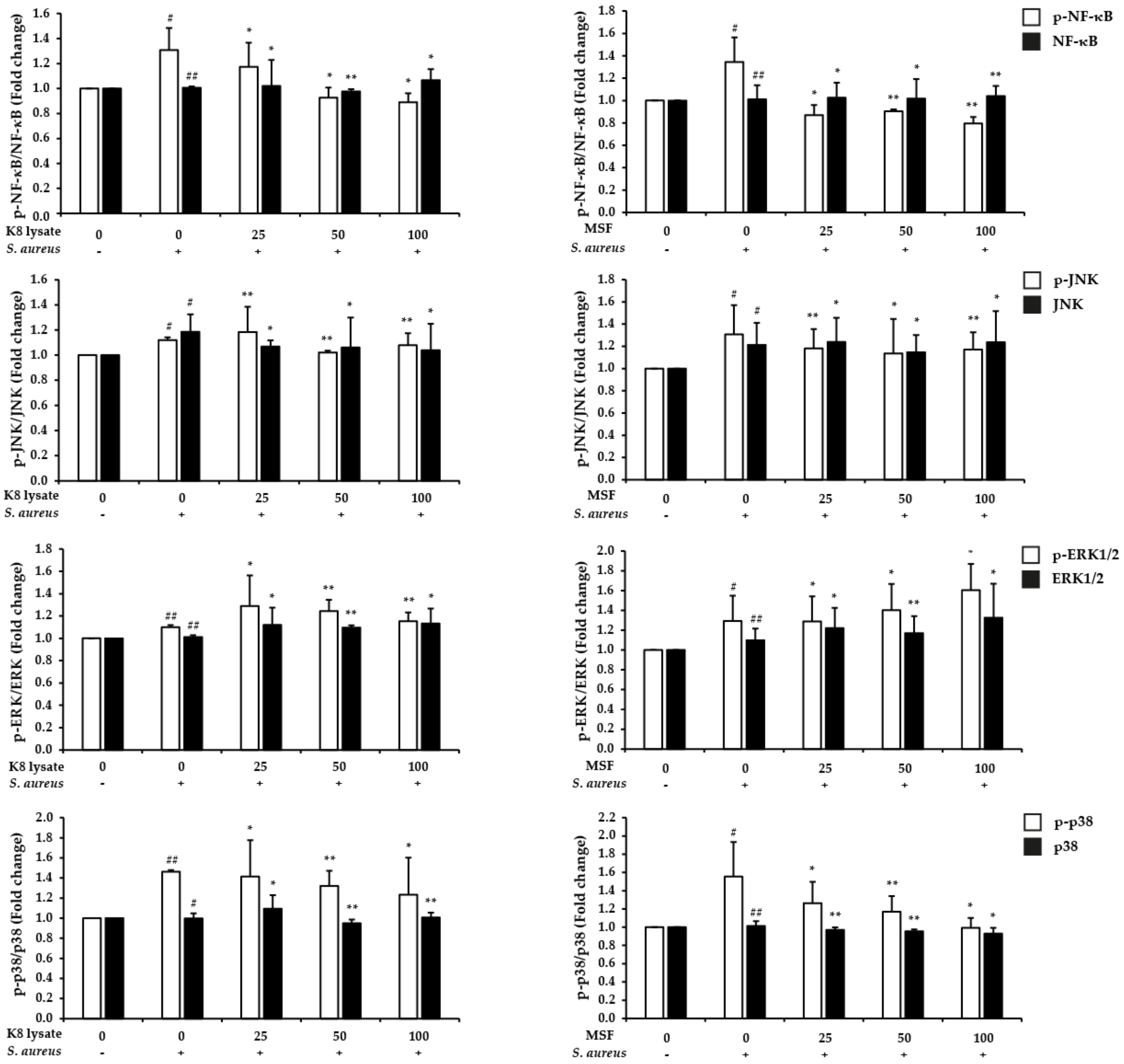

Figure S5. MSF treatment suppressed the *S. aureus*-induced inflammation in THP1 through NF-κB and p38 signaling pathways. THP1 cells were pre-treated for 1 h with the indicated concentration of MSF and K8 lysate and incubated with heat-killed *S. aureus* for 24 h. ImageJ was used to get the quantification results of western blot analysis. $^{\#\#}p < 0.01$, $^{\#\#\#}p < 0.001$ vs. control. $^{*}p < 0.05$, $^{**}p < 0.01$ vs. model.

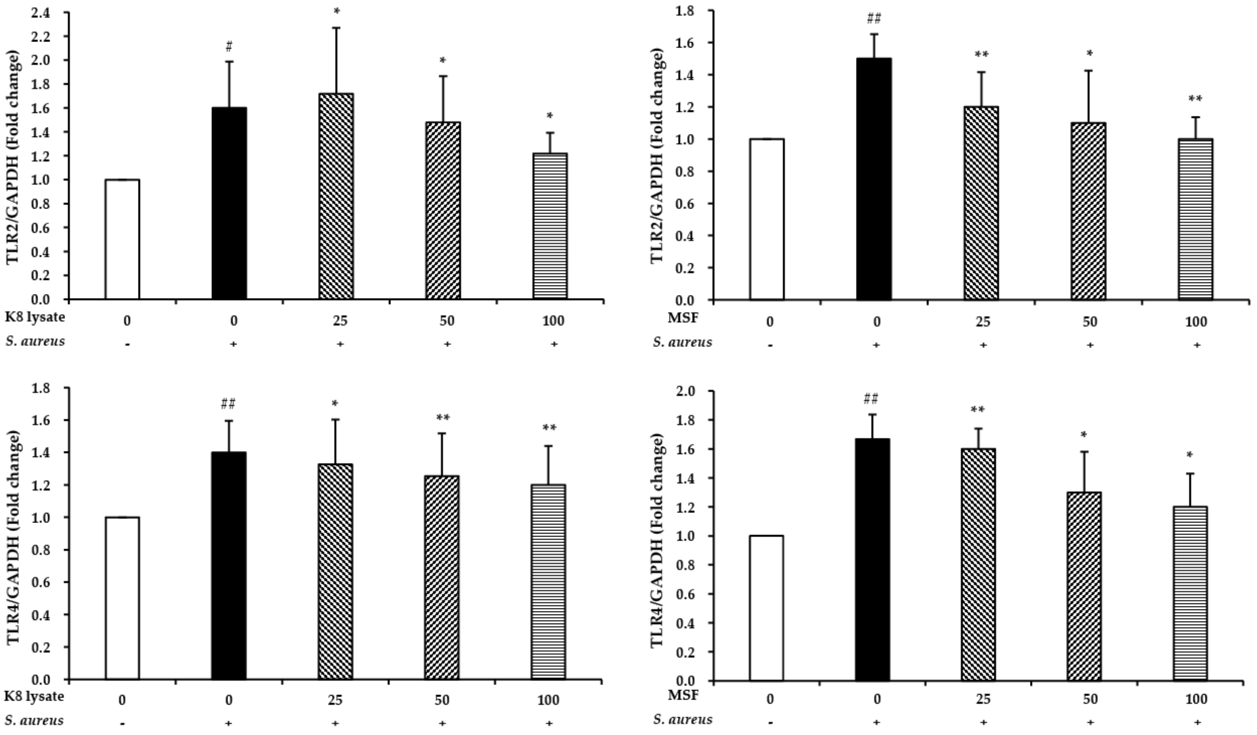

Figure S6. MSF treatment influenced the expression of TLR2 and TLR4. ImageJ was used to get the western blot quantification from western blot manner. $^{\#\#}p < 0.01$, $^{\#\#\#}p < 0.001$ vs. control. $^{*}p < 0.05$, $^{**}p < 0.01$ vs. model.
